# Troubles in the Pocket: Evidence- and Experience-Based Multidisciplinary Expert Consensus Recommendations for Implantable Pulse Generator Placement and Management of Associated Inconveniences

**DOI:** 10.1007/s11916-026-01476-6

**Published:** 2026-04-18

**Authors:** Linda Kollenburg, Inge Arnts, Caro Edelbroek, Colette Milliner, Georgios Matis, Alaa Abd-Elsayed, Jan Willem Kallewaard, Erkan Kurt

**Affiliations:** 1 Department of Anesthesiology, Pain and Palliative Medicine, Radboud University Medical Center, Geert Grooteplein Zuid 10, Nijmegen, 6525 GA The Netherlands; 2 Department of Anesthesiology, Rijnstate Hospital, Wageningseweg 213, Arnhem, 6825 GA The Netherlands; 3 Department of Functional Neurosurgery and Stereotaxis, Krankenhaus Merheim, Walderstraße 25, 51109 Cologne, Germany; 4 Department of Chronic Pain & Spasticity Unit - Neuromodulation, Hygeia Hospital, Sokratous 5-7, Athens, 145 62 Greece; 5 Department of Anesthesiology, UW Health University Hospital, 600 Highland Avenue, Madison, WI 53792 USA; 6 Department of Anesthesiology, Amsterdam University Medical Center, Meibergdreef 9, Amsterdam, 1105 AZ The Netherlands; 7 Department of Neurosurgery, Radboud University Medical Center, Geert Grooteplein Zuid 10, Nijmegen, 6525 GA The Netherlands; 8 Department of Pain, Sint Maartenskliniek, Heinrich Heineplein 10, Nijmegen, 6532 BX The Netherlands

**Keywords:** Implantable pulse generator, IPG, Inconveniences, Neuromodulation, Recommendations, Guidelines

## Abstract

**Purpose:**

More than half of patients treated with neuromodulation experience inconveniences related to the implantable pulse generator (IPG). Despite their significance, there is an absence of standardized guidelines and recommendations minimizing and/or preventing these issues, reinforcing the continuous burden posed by IPG-related inconveniences. This study aims to provide evidence- and experience-based recommendations for preoperative counselling, implantation site, surgical technique, and management strategies, improving IPG-related inconveniences, by combining a comprehensive literature review with insights gathered from global expert meetings on the topic.

**Methods:**

In this evidence-and experience-based consensus study, a literature analysis was performed using the PubMed, MEDLINE, and EMBASE databases. Experts’ insights on the topic were systematically collected during various expert-based meetings. Recommendations were developed through an integration of literature evidence and clinical expertise, with the aim of preventing and reducing IPG-related inconveniences. The level of evidence for each recommendation has been assessed using the GRADE system and the degree of consensus was evaluated using an independent expert-analysis.

**Results:**

A total of 26 articles, published between 2001 and 2024, were included in the literature search. Twenty-six recommendations were formulated. Outcomes emphasized the importance of thorough preoperative counseling, strategic implant site selection, well-considered surgical technique, and tailored postoperative management in reducing the incidence and severity of IPG-related issues. The level of evidence was low to moderate, while the degree of consensus was high for most of the recommendations proposed in this study.

**Conclusions:**

Given the impact of IPG-related inconveniences, clear and practical recommendations are essential for consistent and effective neuromodulation practice.

**Graphical Abstract:**

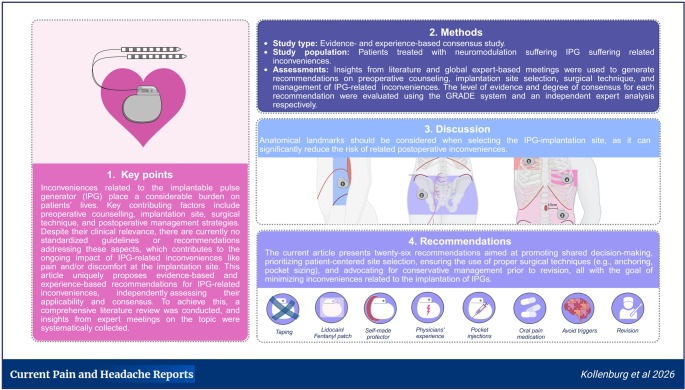

## Introduction

Implantable Pulse Generators (IPGs) were first introduced to the field of neuromodulation in the late 1960s, marking the beginning of a transformative era [1]. Since then, they have undergone continuous innovation, allowing the IPG to accommodate diverse and customizable stimulation protocols. Despite these innovations, ≥ 50% of patients encounter IPG-related inconveniences, which are defined as any difficulties, discomforts, or drawbacks experienced by patients due to their presence, function, or maintenance of the IPG. Common IPG-related inconveniences include pain (26% of patients), clothing discomfort (52% of patients), and psychological distress like embarrassment about the implant (10% of patients) [2]. Such inconveniences can have a considerable negative impact on patients’ quality of life (QoL) [2]. In patients with chronic pain, it is suspected that increased neural sensitization contributes to IPG-related site pain and/or discomfort [3, 4]. Important determinants of IPG-related inconveniences include preoperative counselling, surgical implantation techniques, site selection, and effective management strategies [5]. Despite their significance, standardized guidelines addressing these factors remain absent, thereby causing the persistence of IPG-related inconveniences in most patients [6]. The restricted standardization and clinical awareness on this topic emphasize the importance of research advocating for clinical guidelines aimed at preventing and/or managing IPG-related inconveniences. Following the recent publication of a pioneering article highlighting the significance of IPG-related inconveniences and the importance of their prevention and management [2], there has been a surge of global interest and ongoing discussions on this topic. The current study aims to integrate literature-based evidence with valuable insights from recent global expert meetings on IPG-related inconveniences into practical recommendations regarding preoperative counselling, implantation site selection, surgical technique, and management strategies, to minimize IPG-related issues. This article represents a novel contribution to the field due to the lack of established guidelines on managing and/or preventing IPG-related inconveniences and its interdisciplinary approach, incorporating perspectives from physicians, nurses, researchers and patients to optimize recommendations and improve clinical outcomes of neuromodulation.

## Methods

The goal of this evidence-and experience-based consensus study is to form and evaluate recommendations on: (1) preoperative counselling, (2) IPG- implantation site, (3) surgical technique, and (4) management strategies. To achieve this goal, a comprehensive literature review was integrated with a systematic data collection process during expert-led meetings, which specifically focused on identifying the significance of IPG-related inconveniences and exploring effective strategies for their prevention and management.

### Literature Analysis

A literature search was performed using the PubMed, MEDLINE and EMBASE databases. For the literature analysis, the following search string was used: (’neuromodulation’ OR ‘brain stimulation’ ‘occipital nerve stimulation’ OR ‘spinal cord stimulation’ OR ‘deep brain stimulation’ OR ‘motor cortex stimulation’) AND (‘IPG’ OR ‘implantable pulse generator’ OR ‘pocket’) AND (‘inconveniences’ OR ‘issues’ OR ‘adverse events’ OR ‘counselling’ OR ‘counselling’ OR ‘implantation site’ OR ‘site’ OR ‘placement’ OR ‘surgical technique’, OR ‘management’). Studies were included in this review if they met the following criteria: published in English, classified as original research articles or relevant review articles, and investigating strategies to prevent and/or manage IPG-related inconveniences as either primary or secondary outcomes. Studies were excluded if they were published in languages other than English, included study populations of non-human species, or lacked sufficient detail regarding the occurrence of IPG-related inconveniences and the strategies to prevent and/or manage them. In addition, reports were also manually searched using the search function in Google Scholar, as well as reviews or references cited within the articles. The current analysis did not include articles solely used to find additional studies. Each study analyzed within this literature search is viewed by two independent researchers (LK and EK) to ensure consensus and the quality of the articles included. In the case of disagreement, additional reviewers (IA, CE, CM, GM, AE, and JK) were consulted. Two reviewers collected data from each report (LK and EK).

### Expert-based Meetings

To develop recommendations for the management and prevention of IPG-related inconveniences, expert insights were collected during specialized international meetings. The main topic of these meetings was the management of IPG-related issues, particularly how best to address discomforts and challenges faced by patients. The expert meetings included a diverse audience, consisting of physicians, researchers and nurses, across various locations and events: ‘’*Benelux Neuromodulation Meeting*,* Antwerp (A)(January 6*,* 2024) with ± 80 participants’’*, *‘’Nurse Training*,* Boston Scientific*,* Utrecht (B)(June 13*,* 2024) with ± 30 attendees’’*, ‘’*Educational Training*,* Saluda Medical*,* Breda (C)(November 15*,* 2024) with ± 15 attendees’’*, ‘*’Educational Training*,* Saluda Medical*,* Scheveningen (D)(June 20*,* 2025) with ± 60 attendees’’*, ‘*’European INS*,* Istanbul (E)(May 23*,* 2025) with ± 20 participants’’*, ‘*’International Neuromodulation Society (INS) Conference*,* Vancouver (F)(May 12*,* 2024) with ± 18 participants’’*. During all events (A-F), experts were encouraged to share their insights and suggestions regarding IPG-related inconveniences. In a subset of events (B-D), systematic data collection was applied, while in others, where this was not feasible (A, E, F), insights were obtained and recorded orally during the meetings to gain broader understanding of the topic and strategies for managing IPG-related inconveniences. During systematic data collection, participants were asked to write down their suggestions/recommendations in an online form, alongside their years of experience in the field of neuromodulation and profession. All participants with experience in neuromodulation who actively contributed to the international meetings on this topic (A-F), sharing effective approaches for preventing and/or managing IPG-related inconveniences, constituted the expert panel. Experts were required to have at least two years of experience in the field of neuromodulation and recommendations were solely included if independently supported by at least two experts.

### Evidence Evaluation

Insights derived from literature and expert-based meetings were combined into recommendations, and the overall level of evidence for each was evaluated using the GRADE (Grading of Recommendations, Assessment, Development, and Evaluation) methodology [7]. The certainty of evidence was categorized as ‘*high*’, ‘*moderate’*, ‘*low*’, or ‘*very low’* (Table 1). Afterwards, recommendations were formulated into statements. These statements were combined into a questionnaire, which was created and shared with neuromodulation experts using GRADEpro software (available at https://gradepro.org/) and google forms (https://docs.google.com/forms/u/0/). In the questionnaire, experts were asked to indicate their level of agreement with each statement by selecting one of the following options: *‘Strongly agree*,’ ‘*Agree*’, ‘*Neutral*’, ‘*Disagree*,’ *‘Strongly disagree*,‘. They were also given the opportunity to provide additional comments via the ‘*Other*’ option in the questionnaire, allowing them to explain specific choices, suggest additions, or respond to individual statements. Experts were required to complete this process independently and in a blinded manner, without knowledge of the responses from others and/or outcomes from the literature search and expert-based meetings. To ensure both expertise and independence in participants’ assessments, responses were kept confidential, with participants unable to view others’ answers. The questionnaire invitation also included strict eligibility criteria, such as a minimum of two years of professional experience and no prior attendance at the aforementioned meetings mentioned under ‘*Expert-based meetings’*, in order to preserve objectivity and prevent any potential bias. To visually represent the level of agreement among experts for each recommendation/statement in the questionnaire, a color-coded scheme was applied using the colours; green (high consensus), blue (moderate consensus), pink (low consensus) and red (limited or no consensus).

**Table 1:**
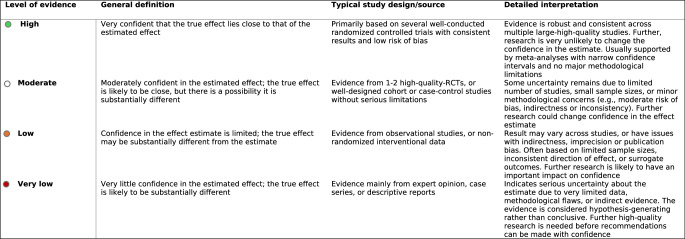
Certainty level of evidence GRADE system

### Ethics statement

The studies involving humans were approved by The Medical Review Ethics Committee region Arnhem-Nijmegen. The studies were conducted in accordance with the local legislation and institutional requirements. The Medical Review Ethics Committee region Arnhem-Nijmegen concluded that this study was not subject to the Medical Research Involving Human Subjects Act (CMO Oost-Nederland; file number: 2025–18626. This study was performed according to the Dutch law and Ethical Principles for Medical Research Involving Human Subjects, outlined in the World Medical Association’s Declaration of Helsinki revised in 2013.

## Results

Based on the insights derived from the literature review and expert discussions, 26 recommendations were developed (Table 2).

### Literature Search

**Fig. 1:**
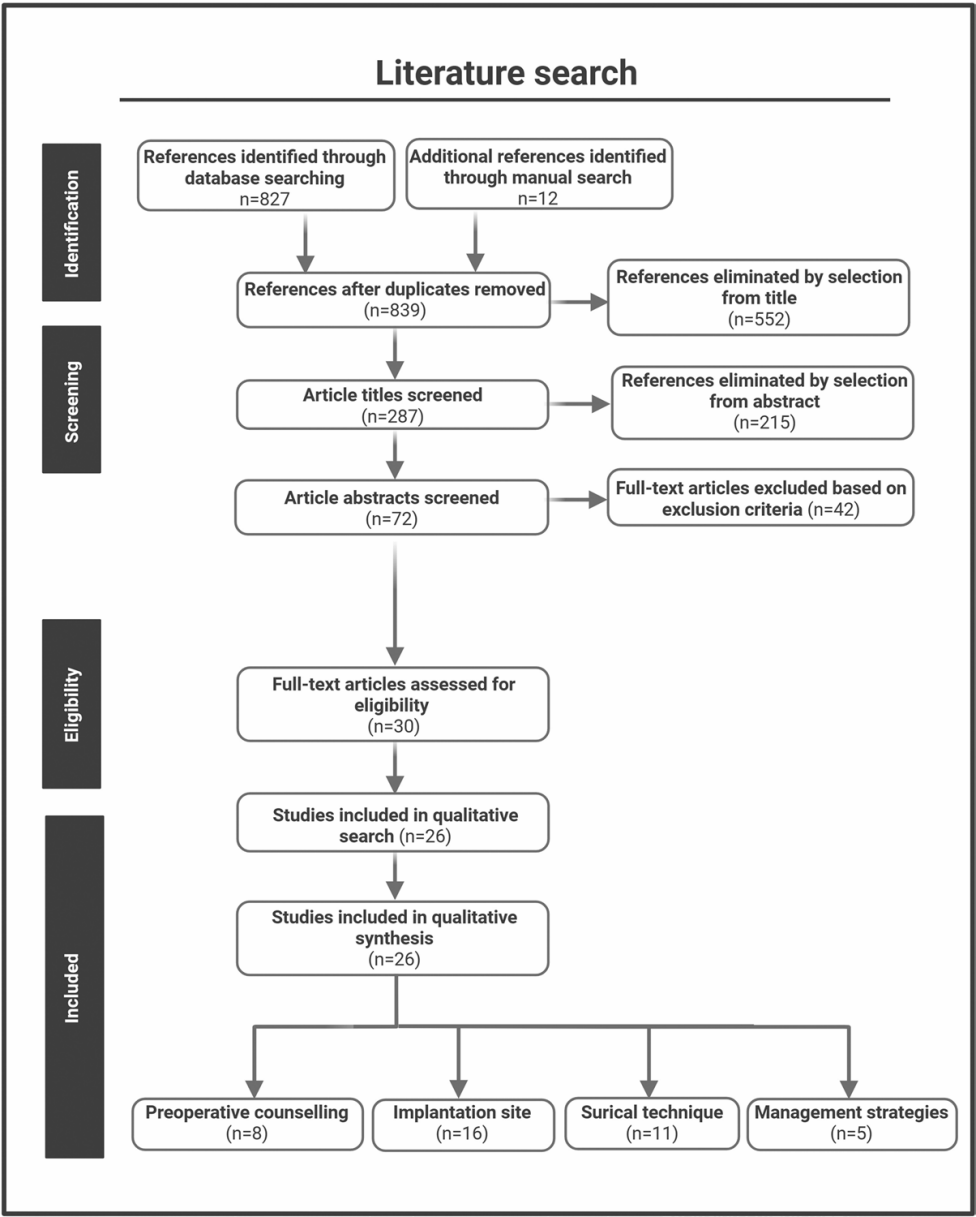
**Literature search.** The literature search was reviewed to analyze preoperative counseling, implantation site, surgical technique, and management strategies for IPG-related inconveniences. Created in BioRender. Kollenburg, L. (2025) https://BioRender.com/ bclqzkl. *Please note that the column ‘studies included in qualitative synthesis’ reflects the total number of articles analyzed within this literature review, with some being listed in more than one category in case multiple factors were covered in the same article

A total of 26 articles, published between 2001 and 2024, are included in the current analysis on preoperative counselling, implantation site and surgical implantation of the IPG, and management strategies for related inconveniences (Fig. 1).

### Preoperative Counselling

Several studies have explored the impact of preoperative counselling in patients undergoing IPG implantation. Kurt et al. examined IPG-related disruptions in spinal cord stimulation (SCS) patients and suggested that preoperative counselling could help set realistic expectations, promoting active patient involvement and control. This approach may improve patient satisfaction despite IPG-related challenges [2] (Table 2). This theory is supported by literature indicating that patient education is critical in optimizing outcomes of neuromodulation therapies. Authors emphasize that involving patients in decision-making regarding IPG placement is essential [8, 9]. Others recommend taping a dummy IPG, or an object of similar size, such as a box of dental floss, onto different areas of the patient’s body to allow them to experience how various placement sites might feel [9, 10]. In addition, it is recommended to mark the intended IPG location while the patient is in a standing or sitting position [11] (Table 2). Preoperative counselling has been shown to support informed decision-making, giving patients a better sense of how the placement may impact their movement and daily activities [9–11]. Further, data highlights the presence of variable perspectives regarding the IPG. Whereas patients often concentrate on factors affecting QoL (e.g. ability to engage in social and daily activities), scientists and physicians usually tend to focus on technical aspects (e.g. duration of the surgery, chances for revision and technical issues, requirement of additional leads, and patient positioning) [2, 6, 12, 13] when it comes to choosing the IPG-implantation site (Table 2). It is noted that both perspectives should be combined in a preoperative counselling setting to ensure shared decision-making and improve IPG-related inconveniences [2].

**Table 2:**
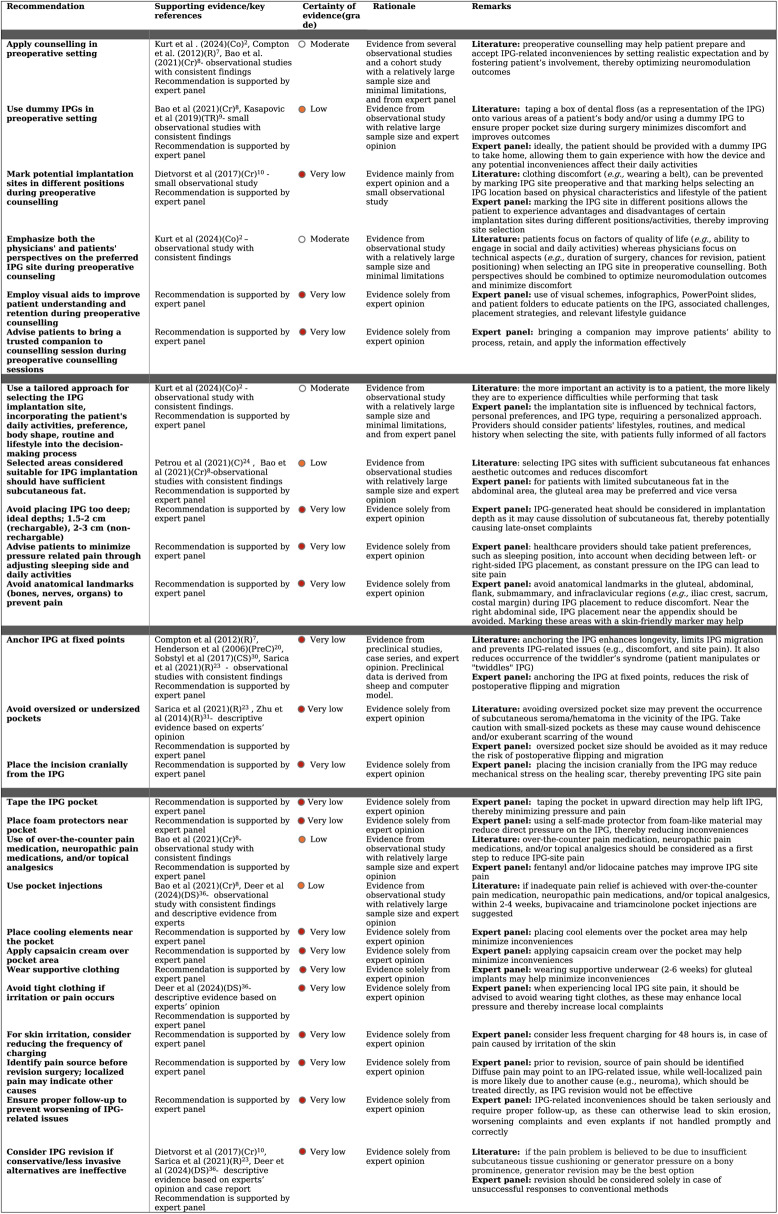
Assessment of certainty level for recommendations for IPG placement and management of related inconveniences

### Implantation Site

**Fig. 2:**
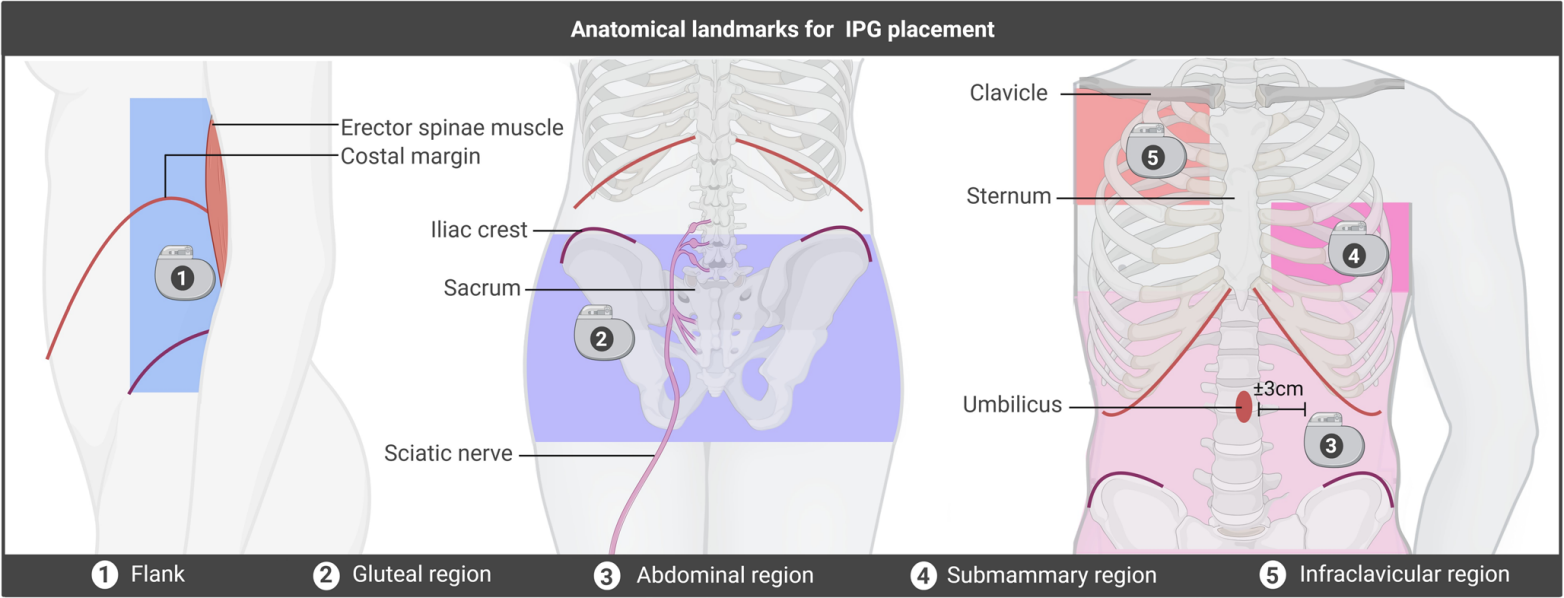
**Anatomical landmarks for IPG placement.** Overview of anatomical landmarks that should be avoided for IPG placement in various regions, to minimize IPG-related inconveniences. (**1**) Flank: costal margin, erector spinae muscle, iliac crest; (**2**) gluteal region: iliac crest, sacrum, sciatic nerve; (**3**) abdominal region: costal margin, iliac crest, and umbilicus; (**4**) submammary region: sternum; (**5**) infraclavicular region: clavicle, sternum. Created in BioRender. Kollenburg, L. (2026) https://BioRender.com/ ik8v3s0

IPGs can be implanted at various anatomical sites depending on the type of neuromodulation therapy [14]: SCS (flank, abdominal, lower back, gluteal) [2, 6, 14], deep brain stimulation (DBS)(infraclavicular, submammary, subpectoral) [3, 15, 16], motor cortex stimulation (MCS)(infraclavicular), or occipital nerve stimulation (ONS)(infraclavicular, abdominal, buttock) [17, 18] (Fig. 2; Table 3). Numerous studies have evaluated the advantages, drawbacks, and complications associated with different IPG sites (Table 3). In SCS, the gluteal region is often preferred due to lower complication and reoperation rates, reduced discomfort, shorter surgery times, and facilitated lead tunnelling, potentially lowering the risk of lead migration [2, 13, 25] (Table 3). Further, data suggests that abdominal implants may disrupt magnetic fields in some environments, leading to unintended IPG shutdown, while flank placement may reduce revision rates [6, 9, 13](Table 3). Authors emphasize that sufficient subcutaneous fat should be present in either of these regions to ensure proper IPG protection and reduce related inconveniences [9] (Tables 2 and 3). Submammary placement is suggested for thin patients or those with prior mastopexy, providing better soft tissue coverage and reduced site pain [29]. While some studies report that implantation site influences lead strain and pocket discomfort [6, 8, 12, 23], others find no significant association in SCS [3, 9], highlighting the importance of proper anchoring, strain relief, and patient education [3, 8] (Tables 2 and 3). For ONS, infraclavicular and buttock regions are favoured over the abdominal area due to lower adverse event rates [20, 27]. Literature describes that abdominal implants require longer tunnelling and patient repositioning, increasing lead strain and risk of migration [20, 27]. A lateral approach allows infraclavicular placement, reducing pocket stress, though cosmetic concerns may arise [27] (Table 3). In DBS, suboptimal IPG positioning is linked to increased complications [26]. The infraclavicular site is commonly used in DBS, due to lower rates of discomfort and other inconveniences [22], but thin patients may benefit from axillary or submammary placement for aesthetic and comfort reasons [28] (Table 3). With regard to submammary implantation in a subglandular or subfascial pocket, it is associated with reduced site pain, improved anchoring, and better cosmetic outcomes, though it may be more technically demanding and time-consuming [22]. Subpectoral or transaxillary subpectoral placement further enhances aesthetics and reduces discomfort, though surgery time and post-operative pain may increase [19, 21, 22, 24, 26] (Table 3). For overweight patients, suprascapular placement can improve fixation and reduce inconveniences [30]. Skull implantation has been reported but carries infection risks, making the chest wall generally preferable [26, 29]. Overall, while IPG site selection varies by therapy, patient anatomy, and surgical considerations, factors such as lead anchoring, strain relief, tissue coverage, and patient education often play a more critical role in minimizing complications and improving outcomes than site alone. Literature on IPG placement in MCS remains limited.

**Table 3:** IPG-implantation sites for spinal cord stimulation, occipital nerve stimulation and deep brain stimulation

Authors (year)	Study design	Diagnosis (sample size)	age (years)	Neuromodulation technique	Ipg-implantation site	Remarks
Scheepens et al. (2001) [12]	CT	Urine-incontinence (22), urgency–frequency syndrome (6), urinary retention (9), pelvic–pain syndrome (1), fecal incontinence (1)	51 (33–72)	SCS	Gluteal- or abdominal region	**Gluteal**: reduced surgery time (1 h), no patient repositioning, shorter subcutaneous tunnelling, only two incisions necessary, lower reoperation rate **Abdominal**: disruption of magnetic field
Garg et al. (2010) [19]	CR	Parkinson’s disease (1)	70	DBS	Upper back	**Suprascapular**: reduced extension wire fractures, especially suitable for overweight females with loose subclavicular subcutaneous tissue **Skull implant**: enhanced risk of cerebral infections
Trentman et al. (2010) [20]	T	N/A	N/A	ONS	Infraclavicular region	**Infraclavicular**: cosmetically less desirable **Gluteal**: enhanced stress on system, relative to abdominal IPG site
Son et al. (2012) [21]	C	Movement disorders (38)	Infraclavicular: 65 (± 7.53), subpectoral: 55.46 (± 15.37)	DBS	Infraclavicular, subpectoral region	**Subpectoral transaxillary**: improved cosmetical results, less discomfort and morbidity related to erosion and infection
Hayek et al. (2015) [18]	LR	Primary lateral sclerosis (203), CRPS (68), small fiber neuropathy (21), abdominal/pelvic pain (15), lumbosacral radial syndrome (14), other neuropathic pain syndromes (24)	53.6 (± 14.9)	SCS	Paraspinous- or upper gluteal region	**Paraspinous**: higher rates of related discomfort relative to upper buttock placement
Hong et al. (2015) [22]	CR	N/A (1)	40	SCS	Submammary region	**Submammary**: enhanced soft tissue coverage, decreased pain at the generator site, and a low rate of complications
Sharan et al. (2015) [23]	CT	Chronic migraine (157)	N/A	ONS	Upper buttock, abdomen, infraclavicular, or lower axilla	**Infraclavicular**: lower AE incidence rate
White-dzuro et al. (2017) [24]	T	Parkinson’s disease (165), essential tremor (98), other diagnoses (38)	62.0 (± 11.6)	DBS	Subpectoral, infraclavicular region	**Subpectoral**: low complication rate (e.g. infection and wound erosion)
Baranidharan et al. (2020) [25]	C	Multiple (764)	54 ((± 13.0)	SCS	Posterior chest wall, or gluteal, flank, abdominal region	**Posterior chest wall region**: lowest rates of IPG pain and surgical revisions prompted by IPG site pain. **Abdomen**: highest rates of IPG-site pain
Bao et al. (2021) [8]	C	PSPS (402), CRPS (183), chronic neuropathic pain (200)	46.86 (± 1.06)	SCS	Flank or gluteal region	**Flank**: preferred site for IPG placement **Gluteal**: suitable alternative in case of insufficient subcutaneous fat in flank area
Choi et al. (2021) [3]	CS	N/A (510)	62.5 (± 13.7)	SCS	Back, gluteal, chest or abdominal region	No correlation between location of IPG placement and incidence or severity of site pain. The most important factor for IPG site-associated pain was having a spinal cord stimulator implanted as compared to a deep brain stimulator, or sacral nerve stimulator.
Mehta et al. (2021) [5]	C	N/A (330)	57.54 (± 13.25)	SCS	Flank or gluteal region	**Flank**: less revision surgeries **Gluteal**: revision more frequent due to site pain Neither IPG site was more likely than the other to require revision surgeries
Petrou et al. (2021) [26]	CR	Parkinson’s disease (2)	64 (61–67)	DBS	Submammary region	**Submammary**: improved cosmetic results, lower complication rates and post-operative pain, improved anchoring of implant
Sarica et al. (2021) [27]	LR	N/A	N/A	DBS	Variable (e.g. subpectoral, infraclavicular region)	**Subpectoral**: improved cosmetic outcomes, lower rates of discomfort
Yuen et al. (2023) [28]	CR	Essential tremor (1)	60	DBS	Submammary region	**Inferior to pectoralis major fascia**: reduced complication rates associated with silicone implant placement **Subfascial implantation**: improved cosmetical outcomes, reduced AE rates complication rates relative to subglandular plane
Kurt et al. (2024) [2]	C	PSPS type 2 (81)	50.9 (± 10)	SCS	Gluteal- or abdominal region	**Gluteal**: preferred site, lower incidence of shame, improved coverage of area, no repositioning, enhanced MRI compatibility as no extensions are required
AE, adverse event; C, cohort study; CS, cross-sectional study; CR, case report; CT, clinical trial; DBS, deep brain stimulation; LR, literature review; N/A, not available; ONS, occipital nerve stimulation; PSPS, persistent spinal pain syndrome; SCS, spinal cord stimulation; T, technical report						

### Surgical Technique

Several articles report a positive correlation between IPG-related inconveniences and surgeons’ experience [20, 25]. An important contributing factor may be that experienced surgeons are familiar with surgical techniques for IPG placement, thereby possibly preventing and/or reducing related inconveniences. Various studies describe that anchoring the IPG enhances longevity and prevents IPG-related issues like discomfort and site pain [8, 23, 26, 31] (Table 2). Sobstyl et al. recommend placing the IPG in the subfascial/submuscular region while anchoring it with non-absorbable sutures and stitching the pocket to reduce its size, thereby limiting IPG movement, which could otherwise lead to pain and/or discomfort [31]. Similarly, others highlight that oversized IPG pockets should be avoided [32], especially when not fixed by anchoring [26]. In addition, it is highlighted that caution should be taken with a small-sized pocket as it may cause wound dehiscence and/or exuberant scarring of the wound [26] (Table 2). Some evidence suggests that spreading vancomycin powder throughout the IPG pocket during insertion reduces infection, thereby minimizing risks of post-implantation inconveniences such as site pain and skin erosion [26]. Arguments in favour of vancomycin powder use for this purpose: its broad availability, effectiveness, and low costs [33, 34]. Additionally, authors note that antibiotic envelopes, initially designed for cardiac implantable electronic devices, may also help prevent post-operative infections and IPG migration, thereby potentially reducing related inconveniences [26, 35]. It is worth noting that although antibiotic envelopes have not yet been extensively studied in the context of neuromodulation, similar effects to those observed in cardiac pacemakers may be expected, given the comparable size of the IPG and the similarity in implantation sites in specific neuromodulation procedures [26]. Furthermore, Hayek et al. describe that when opting for implantation in the paraspinous area, moving the IPG pocket more laterally is recommended as it reduces discomfort, since the pressure on the trunk muscles is avoided [25]. Further, every effort should be made to minimize scarring during IPG implantation, as several authors have identified it as a significant contributor to post-operative pain, discomfort, and poor cosmetic outcomes, which may lead to feelings of embarrassment or shame [36].

### Management Strategies

Once IPG-related inconveniences cannot be prevented through careful selection of site and surgical technique, alternative management strategies should be considered. Bao et al. recommend the use of over-the-counter pain medication, neuropathic pain medications, and/or topical analgesics as a first step to reduce IPG-site pain [9]. In case inadequate pain relief is achieved within 2–4 weeks, bupivacaine and triamcinolone pocket injections are suggested [9, 37] (Table 2). Furthermore, advising patients on potential behavioral triggers and daily activities contributing to IPG-related discomfort can be valuable in preventing or managing such issues [9]. The authors state that, solely if these conventional strategies fail, revision of the IPG should be considered. While most opt for revision in case all traditional strategies have failed to improve IPG-related inconveniences [11, 37], some suggest that prompt revision should be deemed suitable in cases of discomfort or pain caused by interference with the waistband/iliac crest, or by migration with older IPG models [26] (Table 2; Fig. 4). In such instances, obtaining an x-ray is recommended to assess whether relocating the IPG would be beneficial [9]. In case of a revision due to waistband or iliac crest issues, the IPG should preferably be relocated in a more cranial or caudal direction on the same side [9]. It should be highlighted that revision surgery requires careful consideration due to its invasive nature, elevated risk of infection, and substantial associated costs [22].

**Fig. 3:**
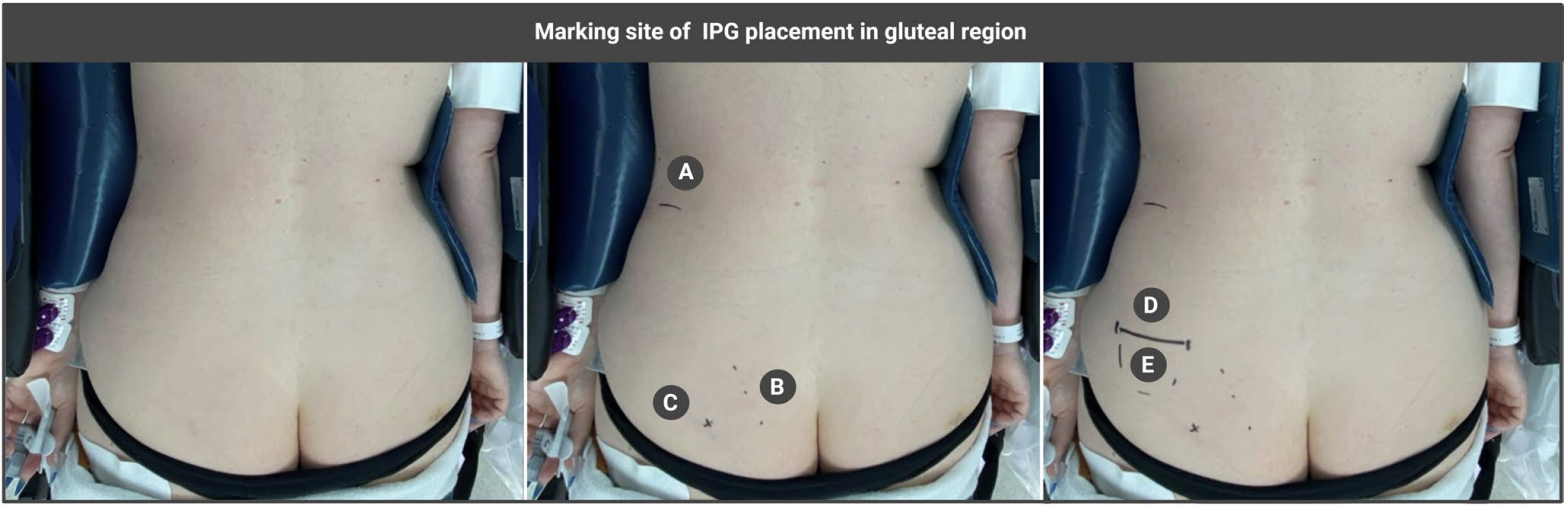
**Marking site of IPG placement in the gluteal region. **Using a skin-friendly marker, an overview of steps to mark anatomical landmarks, pocket, and incision for IPG placement in the gluteal region. Carefully mark the iliac crest (**A**), the lateral border of the sacrum (**B**), and the location of the sciatic nerve, which can be palpated (**C**). Then draw a line to mark the IPG (**D**) incision. The dotted line marks the lower border of the IPG (**E**). Created in BioRender. Kollenburg, L. (2026) https://BioRender.com/4ujgzuo

### Expert-based Meetings

Throughout the expert-based meetings on strategies to prevent and/or manage IPG-related inconveniences, 24 recommendations were formulated based on collected suggestions from 43 participating experts, forming the expert panel (Table 2). The expert panel consists of specialized (functional) neurosurgeons (5)(± 6 years of experience), pain physicians (11)(± 8 years of experience), neuromodulation nurses (19)(± 6 years of experience), and acknowledged researchers in the field of neuromodulation (8)(± 4 years of experience).

### Preoperative Counselling

Experts praise the use of preoperative counselling. It is proposed that health care providers have their patients try everyday movements like bending, with the IPG taped to different sites of the patients’ bodies, to check for site-related discomfort. It was noted that, ideally, patients should receive a dummy IPG to take home to better understand how the device and its potential inconveniences affect their daily activities. Furthermore, the panel recommends using visual schemes, infographics, PowerPoint slides, and patient folders to educate patients on the IPG, associated challenges, placement strategies, and relevant lifestyle guidance. Experts also advise patients to bring a trusted companion to preoperative counselling, as their presence can support the patient in understanding and managing the information more effectively (Table 2).

### Implantation Site

The advisory board supports IPG placement in the gluteal region as this site does not require repositioning during surgery and no extensions are necessary. The expert panel also highlights that IPG placement in the infraclavicular region may be disadvantageous, as it prevents proper screening for breast cancer. Overall, experts emphasize that an optimal IPG implantation site may differ between patients, depending on technical factors, personal preferences (e.g., side on which the patient sleeps), IPG-type (rechargeable/non-rechargeable), as well as its size and shape, which may differ across manufacturers. This underscores the need for a tailored approach in selecting the IPG implantation site. They advise healthcare providers to carefully consider patients’ overall preferences, lifestyles, and routine (e.g., daily and social activities, work, and clothing style) [2], alongside their technical considerations (e.g., ease of surgery) and medical history (e.g., use of insulin injections in abdomen for diabetes), in selecting an IPG site (Table 2). From a technical point of view, the expert panel highlights that body habitus is essential when choosing the IPG implantation site. It is advised to place the IPG in a region covered with sufficient subcutaneous fat, as it improves pocket protection, thereby minimizing discomfort and/or site pain. Experts emphasize that the IPG should not be placed too deep, ideally 1.5–2 cm for rechargeable and 2–3 cm for non-rechargeable devices, as excessive depth may cause charging difficulties, subcutaneous fat dissolution, and late-onset discomfort (Table 2). It is also highlighted that various anatomical landmarks, consisting of bony structures (e.g., iliac crest, costal margin, clavicle, sternum), nerves and organs (e.g., appendix for placement in right abdominal area), should be avoided as these may cause significant pain or discomfort if subjected to pressure (Fig. 2). Experts share that it is essential to mark the anatomical landmarks, IPG pocket, and incision site before implantation, using a skin-friendly marker, so that site selection for IPG placement can be precise (Fig. 3).

**Fig. 4:**
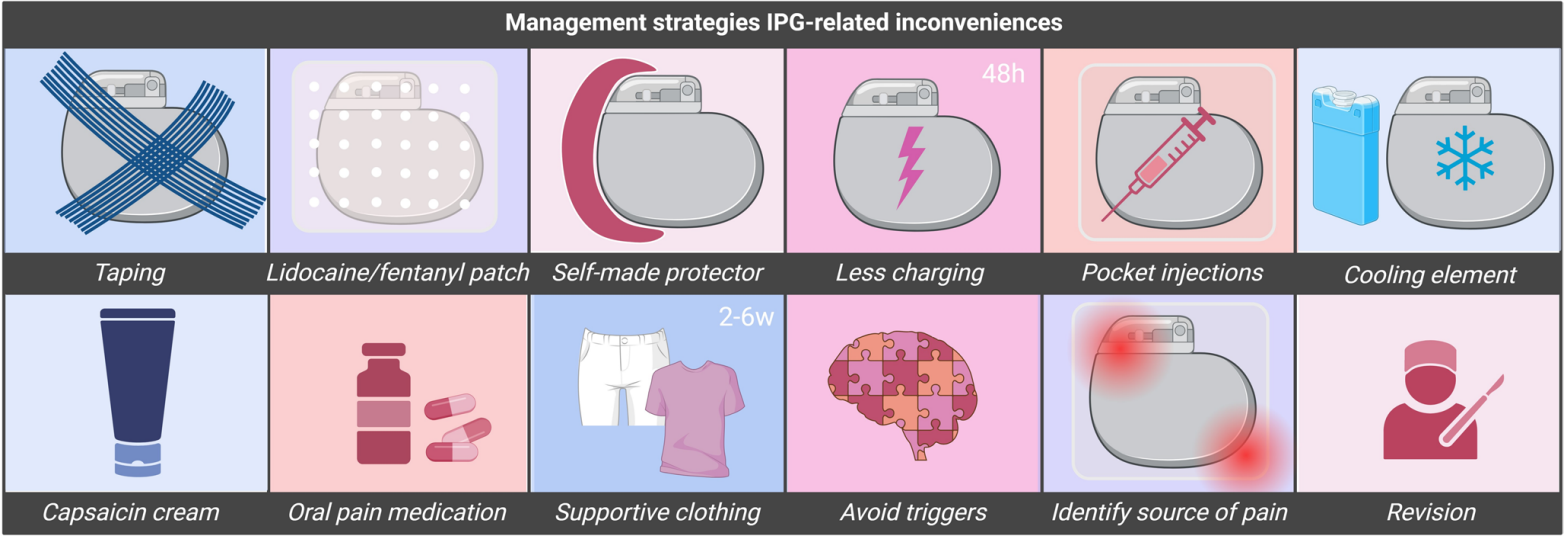
**Management strategies for IPG-related inconveniences. **Overview of management strategies for IPG-related inconveniences. Before considering revision surgery, it is essential to begin with conservative approaches, such as taping, cooling elements, small/fentanyl patches, or custom-made protectors. H, hours; w, weeks. Created in BioRender. Kollenburg, L. (2026) https://BioRender.com/40fyyqv

### Surgical Technique

Furthermore, the advisory board recommends anchoring the IPG at points, preferably on fixed sides of the device using non-absorbable sutures. Experts highlight that the creation of an oversized pocket should be avoided to reduce the risk of postoperative flipping and migration. On the other hand, the pocket shouldn’t be too small, as it will cause local pain immediately from the beginning. The IPG should seamlessly fit into the pocket without the need for excessive force or pressure to secure it. In addition, placing the incision cranially from the IPG is encouraged, thereby reducing mechanical stress on the healing scar and thus preventing pain at the IPG site (Table 2).

### Management Strategies

To address IPG-related inconveniences, taping the pocket in an upward direction is suggested to help lift the IPG, thereby minimizing pressure and pain. Further, experts mention that covering the IPG using a self-made protector from foam-like material, wearing supportive underwear (2–6 weeks) for gluteal implants, or placing a fentanyl or lidocaine patch, cooling element or capsaicin cream over the pocket area may also help minimize inconveniences (Table 2; Fig. 4). In addition, it is shared that in case of severe pain and irritation around the site of IPG implantation, patients should be advised to avoid wearing tight clothes as these may enhance local pressure and thereby increase local complaints. In case of pain caused by irritation of the skin, less frequent charging for 48 h is mentioned to be an appropriate strategy. The panel highlights that revision should be considered solely in case of unsuccessful responses to conventional methods. Moreover, it is proposed that the occurrence and impact of IPG-related inconveniences may vary over the course of a patient’s lifetime (e.g., increased mobility and weight loss may lead to new device challenges). Improved QoL after neuromodulation often increases physical activity, leading to weight loss, particularly in the gluteal region, which may necessitate IPG revision surgery. Such revisions should only be considered after body weight has remained stable for at least three months. Before revision, however, the pain source must be carefully identified: diffuse pain may indicate an IPG-related issue, whereas localized pain often suggests another cause, such as a neuroma, requiring targeted treatment. The panel further emphasizes that IPG-related inconveniences should be taken seriously and followed up promptly to prevent complications such as skin erosion, worsening symptoms, or device explantation.

### Independent Evaluation of Recommendations/Statements

Thirty-five neuromodulation experts were invited to participate in the questionnaire. A total of 30 independent neuromodulation experts responded to the questionnaire, which covered 30 statements, each representing a recommendation proposed in this study (Table 4). Experts had an average of 8.6 (2–25) years of experience. Out of all responders, 50% (15/30) were physician, 6,7% (2/30) were researcher, 37%(11/30) were nurse (practitioners) and 3.3% (1/30) were physician assistant in the field of neuromodulation. Responders originate from various countries including Netherlands (77%, 23/30), USA (13%, 4/30), Greece (3.3%, 1/30), Germany (3.3%, 1/30) and India (3.3%, 1/30). Out of all responders 53.3% (16/30) performs neuromodulation implantations themselves. Consensus levels were high to moderate for all recommendations on preoperative counselling (6/6), most on IPG implantation site (4/5), all on surgical technique (3/3), and the majority on management strategies (10/13)(Table 4). Notably, consensus levels were low for the recommendations on reducing pressure-related pain by adjusting sleeping position and daily activities, for those suggesting that the use of foam protectors is beneficial, and for those indicating that over-the-counter pain medications, neuropathic pain treatments, and/or topical analgesics are effective strategies for managing IPG-related discomfort. Low consensus was also observed regarding the use of pocket injections as a management strategy for IPG-related inconveniences (Table 4). Independent experts added that caution should be taken in patients with excess subcutaneous fat and that consideration of BMI is important when selecting the pocket area. Whether the IPG is rechargeable should be considered when determining the implantation depth. Independent experts also noted that supportive clothing may help minimize pain at the implantation site and that either 0 or 2 fixation points should be preferred when implanting the IPG. Strategies like taping the IPG pocket and using foam protectors were reported to be mostly effective in the early postoperative period. Finally, it was suggested that lidocaine patches should only be prescribed once the patient has recovered (Table 4).

## Discussion

This article uniquely combines evidence-based and experience-based recommendations for IPG related inconveniences, independently assessing their applicability and consensus. Outcomes emphasize the importance of thorough preoperative counseling, strategic implant site selection, well-considered surgical technique, and tailored postoperative management in reducing the incidence and severity of IPG-related issues. Although the level of evidence was low to moderate, with the degree of consensus being high for most recommendations, it is noteworthy that, unlike the available literature, the expert panel proposed a broader range of strategies for management of IPG-related inconveniences, adding the use of tape, foam protectors, supportive clothing, cooling elements and capsaicin cream (Tables 2 and 4). Moreover, the panel adds recommendations on depth of IPG placement, adjusting sleeping side and daily activities to reduce pressure on the pocket, avoidance anatomical landmarks to prevent side pain and placement op the IPG incision, which have not been covered in previous literature (Table 2). The substantial rate of expert-based recommendations achieved in this manuscript may be in part caused by the high prevalence of IPG-related inconveniences among patients receiving neuromodulation [2]. The limited availability of literature-based evidence may stem from the tendency of most studies to prioritize overall pain score improvements as the primary outcome, relegating IPG-related complications to secondary mentions as adverse events, without a focused discussion or section addressing strategies to minimize these issues. The subjectivity of IPG-related inconveniences and lack of standardized assessment of their occurrence may also contributed to scientists being reluctant on thoroughly investigating this topic. Interestingly, the independent review by experts shows that scientific support plays a key role in daily practice, influencing which recommendations are adopted and where consensus has been achieved. To illustrate, recommendations with a moderate to high level of consensus were supported by both scientific evidence and expert discussions, while those with low consensus were based primarily on expert opinion (Tables 2 and 4). The limited availability of data likely accounts for why many recommendations were rated as low or very low, with none categorized as strong (Table 2). This underscores the need for future clinical trials that focus on strategies for managing and/or preventing IPG-related inconveniences.

**Table 4:**
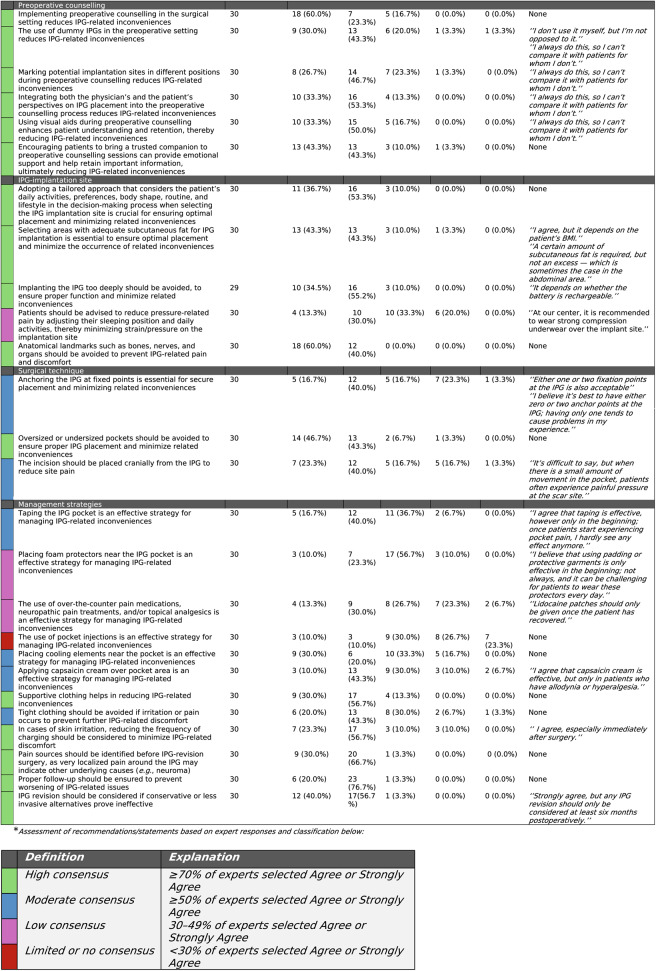
Independent expert evaluation of proposed statements on recommendations to prevent or reduce IPG-related inconveniences

The current study forms a valuable addition to existent guidelines for neuromodulation therapies, proposed by the Neurostimulation Appropriateness Consensus Committee (NACC) in 2024 [37]. While Deer et al. describe recommendations for the mitigation of complications of neurostimulator in a broader context, limited guidelines are proposed for managing IPG-related inconveniences. Their article mainly covers topical therapies for mild symptoms, clothing adjustments, anesthetic injections for scar pain, cushions for low subcutaneous fat, and removal in severe cases [37]. However, it lacks recommendations on preoperative counseling, implantation site, surgical technique, and broader management strategies. The current article fills this gap by presenting additional conservative strategies and introducing many novel, structured recommendations for clinical practice. As these considerations have not been systematically described before, this work represents a valuable contribution to the existing literature. The strengths of this article lie in being the first to provide recommendations specifically focused on the IPG, combining both evidence- and experience-based information across different disciplines (e.g., physicians, nurses, researchers), applying the GRADE system with the level of consensus for the recommendations determined independently by experts. Grading the level of evidence for all recommendations using the GRADE system, improved transparency, clarity, and the reliability of the guidance provided in this manuscript. Further, requesting additional experts to evaluate the proposed recommendations of this manuscript in a blinded and independent manner, minimized confirmation bias while improving practical use, applicability and validity of the recommendations proposed. Aside from these strengths, various limitations should be considered upon interpreting the outcomes of this study. First, results were not stratified by indication, which may influence outcomes, as patients with chronic pain may respond differently to IPG-related inconveniences and management strategies compared to others [3]. Second, some recommendations are based on expert experience rather than quantified evidence. While moderate to high consensus was achieved for most of the suggestions, formal research has not validated the ones marked as having a low level of evidence. Finally, some patients gain excessive weight during the follow-up period, or lose significant weight, leading to a change in the position of the IPG and practice, which turns out to be inconvenient. This has not been investigated in this in the study. Given the limited studies and growing need for practical guidance on this topic, combining evidence with expert insights makes the article highly relevant.

## Key References

- Choi H, Gaiha R, Moeschler SM, Bendel MA, McCormick ZL, Teramoto M, et al.(2021) Factors Associated With Implantable Pulse Generator Site Pain: A Multicenter Cross-Sectional Study. Neuromodulation;24(8):1351-6.
- Baranidharan G, Bretherton B, Richert G, Kay T, Marsh N, Roberts B, et al.(2020) Pocket pain, does location matter: a single-centre retrospective study of patients implanted with a spinal cord stimulator. Reg Anesth Pain Med;45(11):891-7.
- Kurt E, Kollenburg L, Joosten S, van Dongen R, Engels Y, Henssen D, et al.(2024) Preoperative Counseling in Spinal Cord Stimulation: A Designated Driver in Implantable Pulse Generator-Related Inconveniences? Neuromodulation;27(6):1055-61.

## Conclusions

The proposed evidence- and expert-informed recommendations on preoperative counseling, IPG site selection, surgical technique, and strategies for preventing and/or managing IPG-related inconveniences are essential to ensuring consistency and effectiveness in neuromodulation practice.

## Data Availability

Data supporting this manuscript are available from the corresponding author upon reasonable request.

## Abbreviations

DBS: Deep brain stimulation
IPG: Implantable pulse generator
MCS: Motor cortex stimulation
ONS: Occipital nerve stimulation
QoL: Quality of life
SCS: Spinal cord stimulation
